# Indirect effects of COVID-19 on maternal and child health in South Africa

**DOI:** 10.1080/16549716.2022.2153442

**Published:** 2023-01-06

**Authors:** Evelyn Thsehla, Adam Balusik, Micheal Kofi Boachie, Winfrida Tombe-Mdewa, Chodziwadziwa Kabudula, Jacques Du Toit, Kathleen Kahn, Francesc Xavier Gómez-Olivé, Stephen Tollman, Susan Goldstein, Karen Hofman

**Affiliations:** a SAMRC/WITS Centre for Health Economics and Decision Science - PRICELESS SA, School of Public Health, Faculty of Health Science, University of the Witwatersrand, Johannesburg, South Africa; b School of Statistics and Actuarial Science, University of the Witwatersrand, Johannesburg, South Africa; c MRC/Wits Rural Public Health and Health Transitions Research Unit (Agincourt), School of Public Health, Faculty of Health Sciences, University of the Witwatersrand, Johannesburg, South Africa; d Department of Epidemiology and Global Health, Umeå University, Umeå, Sweden

**Keywords:** Immunization, mortality, pregnancy, severe acute malnutrition, pneumonia, diarrhoea

## Abstract

**Background:**

The unfinished burden of poor maternal and child health contributes to the quadruple burden of disease in South Africa with the direct and indirect effects of the COVID-19 pandemic yet to be fully documented.

**Objective:**

To investigate the indirect effects of COVID-19 on maternal and child health in different geographical regions and relative wealth quintiles.

**Methods:**

We estimated the effects of COVID-19 on maternal and child health from April 2020 to June 2021. We estimated this by calculating mean changes across facilities, relative wealth index (RWI) quintiles, geographical areas and provinces. To account for confounding by underlying seasonal or linear trends, we subsequently fitted a segmented fixed effect panel model.

**Results:**

A total of 4956 public sector facilities were included in the analysis. Between April and September 2020, full immunisation and first dose of measles declined by 6.99% and 2.44%, respectively. In the follow-up months, measles first dose increased by 4.88% while full immunisation remained negative (−0.65%) especially in poorer quintiles. At facility level, the mean change in incidence and mortality due to pneumonia, diarrhoea and severe acute malnutrition was negative. Change in first antenatal visits, delivery by 15–19-year olds, delivery by C-section and maternal mortality was positive but not significant.

**Conclusion:**

COVID-19 disrupted utilisation of child health services. While reduction in child health services at the start of the pandemic was followed by an increase in subsequent months, the recovery was not uniform across different quintiles and geographical areas. This study highlights the disproportionate impact of the pandemic and the need for targeted interventions to improve utilisation of health services.

## Introduction

South Africa, as with many countries around the world, has experienced the devastating effects of the Corona Virus Disease 2019 (COVID-19) pandemic. As of July 2022, more than 4 million COVID-19 cases had been reported in South Africa while over a 100 000 deaths were confirmed [1]. There have been reports that this number is an underestimate. Reports by the South African Medical Research Council show that excess mortality between May 2020 and May 2022 was approximately 313 115 [2]. According to the World Health Organization (WHO), the pandemic and lockdown regulations instituted to decrease the spread of the virus have disrupted essential health services around the world [3].

In South Africa, lockdown was instituted on 27 March 2020. Lockdown alert level 5 (between 26 March and 30 April 2020) restricted international and domestic travel, commercial and business activity, events and gatherings in large numbers, and schools and universities attendance [4]. Under lockdown alert level 4 (between 1 May and 31 May 2020), some business activities were allowed to resume and workers allowed to return to work. Subsequent alert levels were adjusted to allow more economic activities whilst social distancing activities were upheld to prevent resurgence of the virus.

While essential services such as health and sales of food were permitted during lockdown level 5 and 4 and subsequent alert levels, the impact of the restrictions on the provision of essential health care services is yet to be fully documented in South Africa. Globally, a number of studies have shown the impact of COVID-19 and the lockdown restrictions on access to different health services [5–11]. In China, inpatient and outpatient health services utilisation decreased significantly after the outbreak of the virus [6]. In Nepal, supply of essential services, immunisation and maternity services were the most affected during the lockdown [7]. In Kenya, the pandemic is said to have reduced inpatient utilisation and increased outpatient department (OPD) visits for sexual violence [8]. In Uganda, the pandemic is said to have indirectly increased pregnancy complications such as eclampsia and stillbirths and premature births due to delayed care-seeking behaviour [12,13].

The indirect effects of the COVID-19 pandemic on health care services through restricted movements could reverse the health gains achieved in recent years especially when it comes to maternal and child health. Maternal and child health form part of the quadruple burden (including communicable and non-communicable disease, injury-related disorders) of disease in South Africa. In recent years, mortality rate in children under five years decreased from 41 in 2012 to 32 per 1000 live births in 2018. The maternal mortality ratio decreased from 164 per 100 000 live births in 2012 to 109 in 2017 [14]. Prior to the pandemic, the goal for government was to reduce under-five mortality rate to less than 25 per 1000 live births and maternal mortality rate to 100 per 100 000 live births by 2025 [15]. The target for the Sustainable Development Goal (SDG) is to reduce maternal mortality to 70 per 100 000 live births.

In South Africa, previous studies have shown the impact of the pandemic on maternal and child health services at the provincial level whilst others have shown the impact at national level [16,17]. These studies, however, were limited to the first 6 months of the pandemic which covered the first wave of COVID-19. It is not yet clear what the impact of subsequent waves has been on utilisation of health care services. In other countries, utilisation of services continued to decline during follow-up months of the pandemic whilst in others there was recovery. Given the mutative nature of the virus and the different lockdown restrictions that were introduced, it is important to document utilisation of routine healthcare services under different waves and lockdown restrictions.

Furthermore, the health impact of the pandemic on different socio-economic groups is not documented. In South Africa, access to maternal health services remains inequitable. Between 2008 and 2012, the gap between wealthiest and poorest quartiles receiving antenatal care increased from 5.7% to 16% [18]. Whilst the economic impact of the pandemic on different socio-economic class has been shown, it is still not clear what the health impacts have been. The aim of this study is to investigate the indirect effects of COVID-19 on maternal and child health by different geographical regions and relative wealth quintiles.

## Methods

This is a quantitative analysis of the indirect effects of COVID-19 on selected maternal and child health indicators in South Africa.

### Data sources

We used data from the district health information system (DHIS2). The DHIS2 captures data on key indicators for routine monitoring and evaluation of healthcare provision in South Africa’s public facilities. Public facilities included fixed and mobile clinics (*customised motor vehicle that travels to communities to provide healthcare services*), community health centres as well as hospitals funded by the government. Approximately 61% of the population access care in public health facilities [19]. Data was collected on a monthly basis from all public facilities in all nine provinces. For this analysis, data from January 2018 to June 2021 were collected using Excel Microsoft Office Software (version plus 2016).

### Variables

The main outcome variables are listed in Table 1. We selected these variables because they are routinely collected to monitor utilisation of maternal and child health services in the public sector. Other variables included the relative wealth index (quintile 1: poorest, quintile 2: poor, quintile 3: middle, quintile 4: wealthy, quintile 5: wealthiest), geography (urban, peri-urban, and rural) and the nine provinces.

**Table 1.** Outcome variables and their definitions.

Variable name	Definition
Full immunization	Immunisation coverage for children under 1 year
Measles first dose	Measles first dose for children under 1 year
Diarrhoea incidence	Incidence of diarrhoea with dehydration in children under 5 years
Diarrhoea deaths	Diarrhoea case fatality in children under 5 years
Pneumonia incidence	Incidence of pneumonia in children under 5 years
Pneumonia deaths	Pneumonia case fatality in children under 5 years
Severe acute malnutrition (SAM) incidence	Incidence of severe acute malnutrition in children under 5 years
Severe acute malnutrition deaths	Severe acute malnutrition deaths in children under 5 years
Facility deaths	Death of children between 12–59 months in facility
First antenatal visit	Antenatal first visit coverage
Delivery	Delivery by women between the age of 15 and 19
Caesarean section	Delivery by caesarean section
Maternal deaths	Maternal deaths in facility

### Data analysis

The impact of the lockdown restrictions on the different outcomes was estimated first by calculating changes in the outcomes across facilities, relative wealth index (RWI) quintiles and geographical areas and provinces. Subsequently, to account for confounding by underlying seasonal or linear trends, we fitted a segmented fixed effect panel model. The analysis was stratified by RWI index, geographic area (urban/rural) and province. The RWI was adopted from Chi et al. (2021) who constructed the index using standardised set of questions from household surveys. Using housing characteristics such as roof material, rooms in house, floor material, and water supply, an RWI was calculated by taking the first principal component of the questions [20]. We adopted this approach because the estimates were more granular making it possible to match each facility to an RWI within a 5 km radius.

The regression took the following general form:

$$y_{ft}=\alpha_{0}+\beta_{1}PreTrend_{t}+\beta_{2}PostCovid_{t}+\overset{11}{\underset{i=1}{\sum }}\gamma_{i}\;Month_{i}+\nu_{f}+\epsilon_{ft}$$

Where $y_{ft}$ is the outcome indicator, e.g., full immunisation, $\alpha_{0}$ is the intercept, t indexes time starting from the beginning of the sample, i.e. January 2018, $PreTrend_{t}$ is a variable reflecting the number of months since January 2018, $PostCovid_{t}$ is the number of months since the first COVID wave (i.e. $PostCovid_{t}=1$ in April 2020, $PostCovid_{t}=2$ in May 2020 and so on), $\beta_{i}$ are the fitted model parameters, $\gamma_{i}$ are the fitted seasonal coefficients and $Month_{i}$ represents a corresponding month indicator (with December as the reference category). $\nu_{f}$ is a facility indicator which represent the unobserved underlying heterogeneity that the fixed effect model caters for, and $\epsilon_{ft}$ is an idiosyncratic mean zero error term. We estimated the impact first from April 2020 to September 2020 and then extended the analysis to June 2021. We used Python (version 3.6) software for data cleaning and analysis.

## Results

Table 2 is a summary of the sample characteristics. A total of 4 956 public health facilities were included in the analysis. Clinics represented the majority (68.0%), while mobiles and hospitals accounted for 17% and 10% of the facilities, respectively. Forty four percent of the facilities were situated in urban areas compared with 46.2% in rural areas. The Eastern Cape Province had the highest number (19.9%) of facilities followed by Kwazulu-Natal province with 17.3% of the facilities.

**Table 2.** Sample characteristics.

Facility characteristics	Frequency (Percentage)
**Sample size**	4 956
**Facility type:**	
Clinic	3372 (68.0%)
Community Health Centre	266 (5.4%)
Hospital	486 (9.8%)
Mobile	832 (16.8%)
**Geography:**	
Peri-Urban	486 (9.8%)
Rural	2289 (46.2%)
Urban	2181 (44.0%)
**Province:**	
Eastern Cape	987 (19.9%)
Free State	354 (7.1%)
Gauteng	518 (10.5%)
KwaZulu-Natal	858 (17.3%)
Limpopo	648 (13.1%)
Mpumalanga	405 (8.2%)
Northern Cape	149 (3.0%)
North West	427 (8.6%)
Western Cape	610 (12.3%)
Relative wealth index quintile*	
Quintile 1: Poorest (−0.79; −0.12)	1146 (23.1%)
Quintile 2: Poor (−0.12; 0.18)	1098 (22.2%)
Quintile 3: Middle (0.18; 0.46)	1020 (20.6%)
Quintile 4: Wealthy (0.46; 0.8)	1004 (20.3%)
Quintile 5: Wealthiest (0.8; 1.34)	688 (13.9%)

*Relative wealth index boundaries in parenthesis.

### Child health indicators: immunization

**Figure 1.**
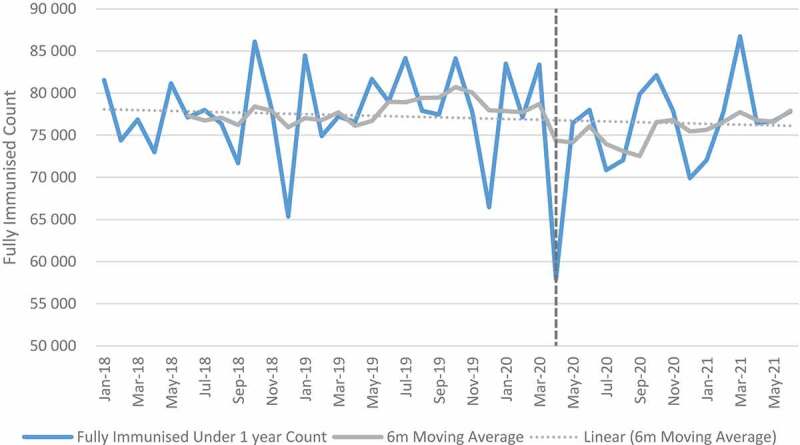
Full immunisation count over time.

**Figure 2.**
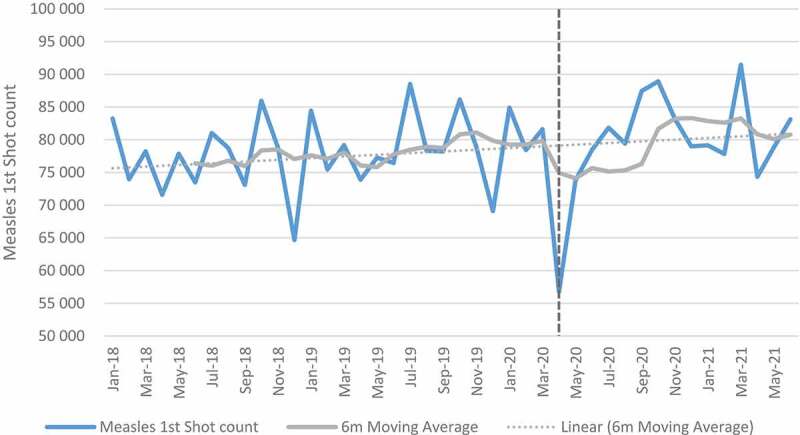
Measles first dose count over time.

Figures 1 and 2 show trends in full immunisation and measles first dose pre- and post-COVID. Full immunisation and measles first dose declined in April 2020 coinciding with level 5 lockdown restrictions. A slight decreasing trend (observing 6-month moving average) was observed for full immunisation in the months that followed even though the numbers increased after April 2020. For the full sample of facilities (Table 3), the mean change for full immunisation in absolute numbers was −5446 between April and September 2020 indicating a −6.99% relative change. The mean change was highest in quintile 5 (−8.8%) and lowest in quintile 1 (−4.1%). The impact was also high in urban (−7.8%) and peri-urban (−8.6%) as compared to rural areas (−5.3%). Mpumalanga province had the highest impact with a mean change of −13.2%. The relative mean change was low in Free State (−1.8%), Limpopo (−1.9%), and the Western Cape (−1.1%).

**Table 3.** Mean changes in immunisation and Measles first dose.

	April 2020 – September 2020				April 2020 – June 2021			
	Fully Immunised		Measles First Dose		Fully Immunised		First Measles Shot	
	Mean pre-COVID-19 (Mean post-COVID-19)	Absolute (Relative Change)	Mean pre-COVID-19 (Mean post-COVID-19)	Absolute (Relative Change)	Mean pre-COVID-19 (Mean post-COVID-19)	Absolute (Relative Change)	Mean pre-COVID-19 (Mean post-COVID-19)	Absolute (Relative Change)
**All facilities**								
All facilities	77 885 (72 439)	−5 446 (−7.0%)	78 089 (76 184)	−1 906 (−2.4%)	77 170 (76 668)	−502 (−0.7%)	77 324 (81 098)	3 773 (4.9%)
**Relative wealth index quintile**								
Quintile_1: Poorest	11 736 (11 254)	−482 (−4.1%)	10 930 (10 821)	−108 (−1.0%)	11 651 (11 430)	−221 (−1.9%)	10 858 (11 245)	386 (3.6%)
Quintile_2: Poor	13 249 (12 374)	−876 (−6.6%)	13 461 (13 516)	54 (0.4%)	13 123 (12 639)	−483 (−3.7%)	13 326 (14 086)	760 (5.7%)
Quintile_3: Middle	11 212 (10 524)	−688 (−6.1%)	11 606 (11 396)	−210 (−1.8%)	11 119 (11 057)	−63 (−0.6%)	11 484 (12 173)	688 (6.0%)
Quintile_4: Wealthy	17 545 (16 270)	−1 275 (−7.3%)	17 733 (17 038)	−695 (−3.9%)	17 372 (17 596)	224 (1.3%)	17 527 (18 522)	996 (5.7%)
Quintile_5: Wealthiest	24 143 (22 018)	−2 126 (−8.8%)	24 359 (23 413)	−947 (−3.9%)	23 906 (23 947)	41 (0.2%)	24 129 (25 072)	943 (3.9%)
**Geography**								
Peri-Urban	5 663 (5 175)	−488 (−8.6%)	5 568 (5 390)	−177 (−3.2%)	5 600 (5 548)	−52 (−0.9%)	5 508 (5 756)	247 (4.5%)
Rural	26 947 (25 516)	−1 431 (−5.3%)	26 755 (26 704)	−51 (−0.19%)	26 705 (25 915)	−790 (−3.0%)	26 505 (27 895)	1 390 (5.2%)
Urban	45 274 (41 748)	−3 527 (−7.8%)	45 767 (44 090)	−1 677 (−3.66%)	44 865 (45 205)	340 (0.8%)	45 311 (47 447)	2 136 (4.7%)
**Province**								
Eastern Cape	9 996 (8 815)	−1 181 (−11.8%)	9 416 (8 770)	−646 (−6.9%)	9 916 (9 344)	−572 (−5.8%)	9 348 (9 264)	−84 (−0.9%)
Free State	3 302 (3 242)	−60 (−1.8%)	3 482 (3 522)	41 (1.2%)	3 287 (3 377)	90 (2.7%)	3 468 (3 671)	204 (5.9%)
Gauteng	18 561 (17 080)	−1 481 (−8.0%)	18 758 (17 791)	−967 (−5.2%)	18 363 (18 747)	384 (2.1%)	18 543 (19 360)	817 (4.4%)
KwaZulu-Natal	18 441 (16 772)	−1 669 (−9.1%)	16 118 (15 319)	−800 (−5.0%)	18 239 (18 338)	99 (0.5%)	15 940 (16 488)	548 (3.4%)
Limpopo	7 836 (7 691)	−145 (−1.9%)	9 688 (10 084)	396 (4.1%)	7 741 (6 861)	−879 (−11.4%)	9 555 (9 949)	394 (4.1%)
Mpumalanga	7 043 (6 112)	−931 (−13.2%)	6 556 (6 501)	−56 (−0.9%)	6 984 (6 885)	−99 (−1.4%)	6 502 (7 333)	831 (12.8%)
North West	4 246 (4 464)	218 (5.1%)	4 685 (4 515)	−169 (−3.6%)	4 220 (4 460)	240 (5.7%)	4 626 (4 972)	345 (7.5%)
Northern Cape	1 005 (891)	−114 (−11.3%)	1 145 (1 043)	−102 (−8.9%)	992 (941)	−51 (−5.2%)	1 131 (1 123)	−8 (−0.7%)
Western Cape	7 457 (7 373)	−84 (−1.1%)	8 241 (8 640)	398 (4.8%)	7 429 (7 716)	287 (3.9%)	8 213 (8 939)	726 (8.8%)

Relative wealth index reported for 5km buffer for facility locations.

When the analysis was extended to June 2021 (Table 3) to include the second wave, the impact was lower with a −0.7% mean change in all facilities. Quintiles 4 and 5 showed a positive mean change while quintiles 1 to 3 were negatively affected. Provinces such as Limpopo (−11.4%), Eastern Cape (−5.8%), and Northern Cape (−5.2%) which are mostly rural also showed a negative impact as compared with other provinces. While the results of the regression analysis were positive at facility level, the increase was not significant. For rural areas and quintiles 2, the decrease was significant while quintile 3 and peri-urban increased significantly (see Table G in Additional File).

For measles first dose, the impact was also negative between April and September 2020 with mean change of −2.4% in all facilities. A positive impact was shown in quintile 2, Free State and Limpopo province. When the analysis was extended to June 2021, the mean change in all facilities increased by 4.9%. Eastern Cape and Northern Cape provinces were the only provinces with a slightly negative change at −0.9% and −0.7%, respectively. The results of the regression analysis were positive but not significant except for quintile 1 (see Table H in Additional File).

### Child health indicators: illness and mortality

**Figure 3.**
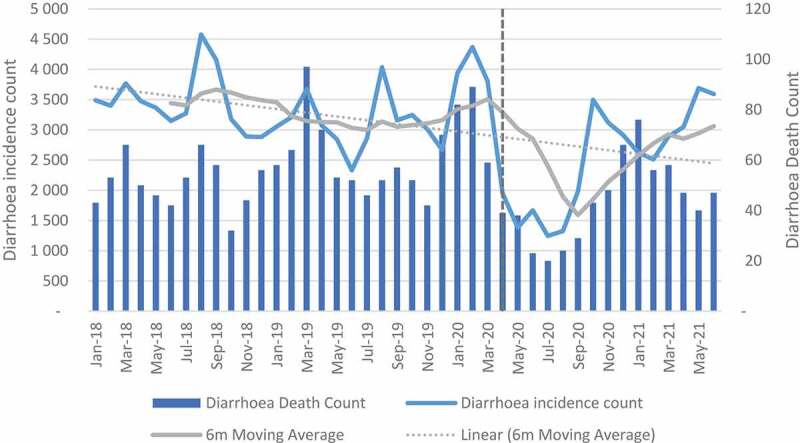
Diarrhoea incidence and death count.

**Figure 4.**
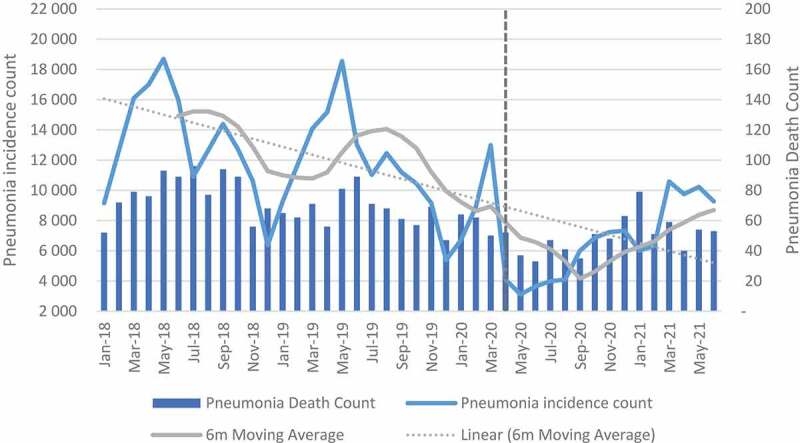
Pneumonia incidence and death count.

**Figure 5.**
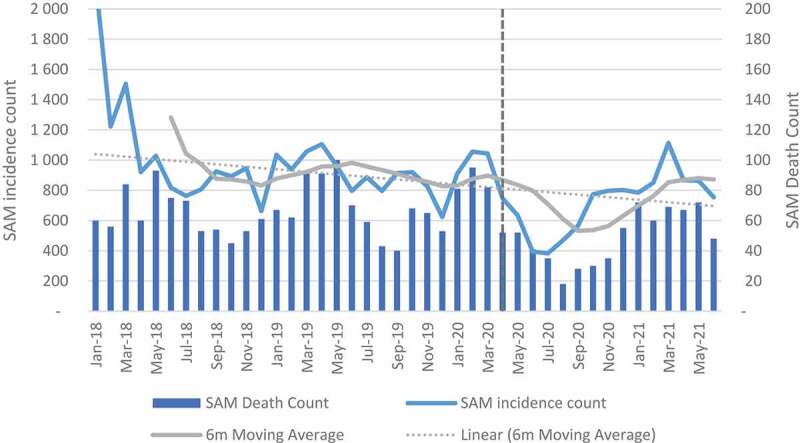
Severe acute malnutrition incidence and death count.

A decreasing trend was observed over time for pneumonia, diarrhoea and SAM incidences (see Figures 3–5). The mean change between April and September 2020 decreased in all facilities by −52.7%, −65.7% and −46.0% for diarrhoea, pneumonia and SAM, respectively (see Table A in Additional file). When the analysis was extended to June 2021, diarrhoea declined by −23.6%, pneumonia by −43.0% and SAM by −26.4%. Northern Cape, Mpumalanga and Eastern Cape were the only provinces with an increase in diarrhoea and SAM when the analysis was extended (see Table B in Additional file). For the regression analysis, the impact at facility level was positive but not significant for pneumonia and diarrhoea. For diarrhoea, a negative significant impact was observed in quintile 1, while quintile 4 and peri-urban showed a positive significant impact. For pneumonia, quintile 4 and peri-urban also showed positive significant impact. The impact on SAM incidence was positive and significant except for quintile 3 which had less incidences when compared with incidence before lockdown restrictions (see Table I – K in Additional File).

Mortality in the 12 to 59 months category was reduced by 32.0% in all facilities between April and September 2020. The highest reduction was observed in quintile 1 (−45.0%), peri-urban (−36.4%) and the Free State province (−66.7%). When the analysis was extended, a decline of −21.2% was observed. Quintile 1 and rural had the highest reduction of approximately 40% and 27.6%, respectively. In the Northern Cape and Free State, mortality was reduced by 33.3% whilst the North West province had the highest reduction (50.0%) (see Table 4). The regression analysis for mortality showed negative and significant impact in quintile 1 and 2 and rural areas.

**Table 4.** Mean changes in 12-59 m deaths in facility.

	April – September 2020		April 2020 – June 2021	
	Mean pre-COVID-19 (Mean post-COVID-19)	Absolute (Relative Change)	Mean pre-COVID-19 (Mean post-COVID-19)	Absolute (Relative Change)
**All facilities**				
All facilities	153 (104)	−49 (−32.0%)	151 (119)	−32 (−21.2%)
**Relative wealth index quintile**				
Quintile_1: Poorest	20 (11)	−9 (−45.0%)	20 (12)	−8 (−40.0%)
Quintile_2: Poor	29 (20)	−9 (−31.0%)	29 (22)	−7 (−24.1%)
Quintile_3: Middle	37 (26)	−11 (−29.7%)	37 (28)	−9 (−24.3%)
Quintile_4: Wealthy	34 (25)	−9 (−26.5%)	33 (30)	−3 (−9.1%)
Quintile_5: Wealthiest	33 (22)	−11 (−33.3%)	32 (26)	−6 (−18.8%)
**Geography**				
Peri-Urban	11 (7)	−4 (−36.4%)	11 (9)	−2 (−18.2%)
Rural	29 (19)	−10 (−34.5%)	29 (21)	−8 (−27.6%)
Urban	112 (78)	−34 (−30.4%)	111 (88)	−23 (−20.7%)
**Province**				
Eastern Cape	21 (14)	−7 (−33.3%)	21 (15)	−6 (−28.6%)
Free State	9 (3)	−6 (−66.7%)	9 (6)	−3 (−33.3%)
Gauteng	31 (21)	−10 (−32.3%)	30 (27)	−3 (−10.0%)
KwaZulu-Natal	29 (24)	−5 (−17.2%)	29 (24)	−5 (−17.2%)
Limpopo	23 (16)	−7 (−30.4%)	23 (18)	−5 (−21.7%)
Mpumalanga	11 (9)	−2 (−18.2%)	11 (10)	−1 (−9.1%)
North West	16 (7)	−9 (−56.3%)	16 (8)	−8 (−50.0%)
Northern Cape	3 (4)	1 (33.3%)	3 (4)	1 (33.3%)
Western Cape	9 (7)	−2 (−22.2%)	9 (8)	−1 (−11.1%)

Relative wealth index reported for 5km buffer for facility locations.

Mortality due to diarrhoea, pneumonia and SAM between April and September 2020 declined by 50.0%, 42.3% and 44.1%, respectively, in all facilities. When the analysis was extended to June 2021, the impact was shown to be less with 22.8%, 30.0% and 26.9% for diarrhoea, pneumonia and SAM, respectively, in all facilities (see Tables C and D in Additional File). The regression analysis for diarrhoea showed a significant decline only in quintile 1. At facility level the impact was positive but not significant. For pneumonia, the impact was positive and significant in quintile 4 and in peri-urban areas. For SAM, the results were positive and significant in all categories except for quintile 3 which was also negative but significant (see Tables I-K in Additional File).

### Maternal indicators

**Figure 6.**
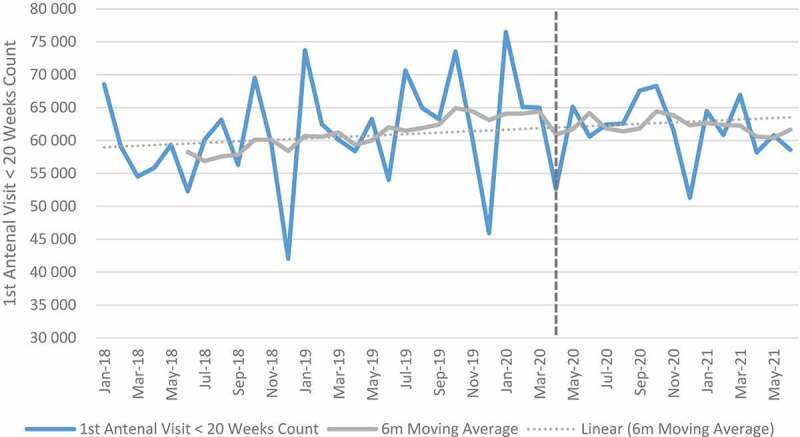
First antenatal visit before 20 weeks.

**Figure 7.**
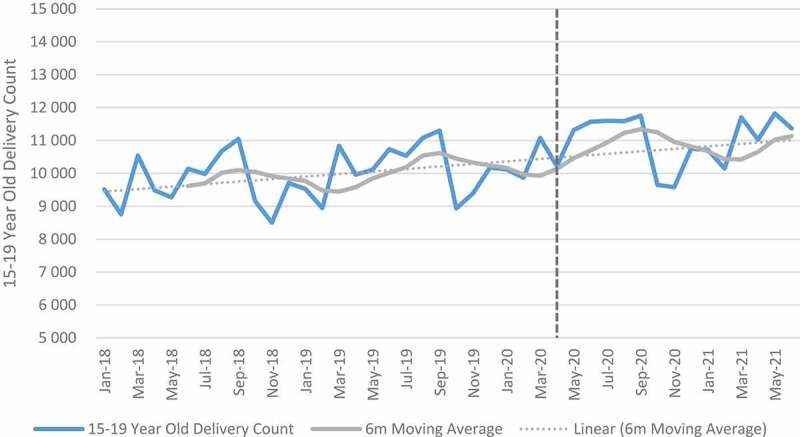
Delivery in facility by 15-19 year olds.

**Figure 8.**
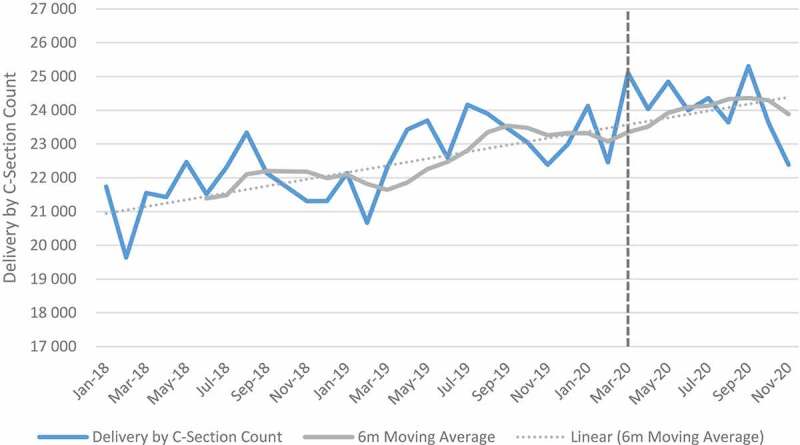
Delivery by C-section.

**Figure 9.**
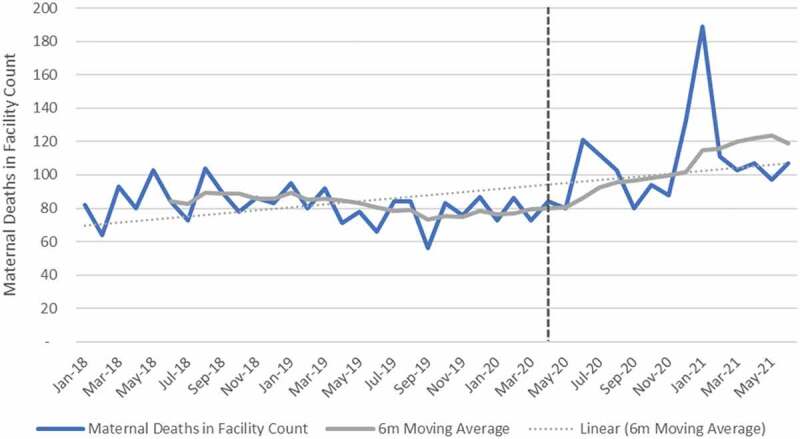
Maternal deaths in facility.

Figures 6–9 shows trends in maternal health indicators pre- and post-COVID-19. Between April and September 2020, the relative change for all maternal indicators included in the analysis was positive when aggregated at the facility level. First antenatal visit, delivery by 15–19-year-olds, delivery by C-section and maternal deaths in facility increased by 0.7%, 13.7% 8.9% and 18.3% respectively. First antenatal visits, however, declined in quintile 4 (−3.2%) and quintile 5 (−8.0%). Urban areas also showed a decline (−5.2%) in first antenatal visit as compared with other geographical areas, which were mostly positive. Eastern Cape, Free-State, Gauteng, Northern Cape, Western Cape and North West showed a reduction in the first antenatal visit. Delivery by C-section also declined in the Free-State. At facility level, an increase of 18% was reported for maternal mortality. When the analysis was extended to 2021, maternal mortality increased by 33%. First antenatal visits increased, while delivery in 15–19-year-olds and delivery by C-section remained positive but slightly less than during the first wave. Antenatal visits declined in Free-State (−7.3%), Northern Cape (−5.5%) and Gauteng (−5.1%) as compared with other provinces that showed an increase. Delivery by C-section also declined in Free-State (−1.3%) and North West province (−0.9%) (see Tables E and F in Additional File). The results of the panel regression analysis were not significant for all maternal health indicators (see Tables L-O in Additional File).

## Discussion

The aim of this study was to investigate the indirect effects of COVID-19 on maternal and child health in South Africa. We first analysed the impact of COVID-19 from April to September 2020. This period covered the first lockdown which had the most restrictions and the first wave which reached peak levels in July 2020. We further extended the analysis to June 2021 to include the second wave that peaked in January 2021.

Our study results showed that at the start of the first lockdown, full immunisation and first dose of measles declined with the highest impact recorded in wealthier quintiles, and in urban and peri-urban areas. The most likely reason for this observation is the movement of individuals and households across provinces and rural and urban areas. In South Africa, the announcement of the first lockdown came with a grace period to allow movement across provinces and between cities and rural areas. According to Posel and Casale (2021), 16% of adults moved into a different household during the first phase of the lockdown, which could explain the change in utilisation of services [21].

In the second period, wealthier quintiles picked up with positive mean changes, while poor quintiles continued to be negative. Rural provinces including Limpopo, Eastern Cape, Mpumalanga and Northern Cape recorded a decline in immunisation compared to the other five provinces where immunisation increased though not to levels similar to pre-COVID period. The change in utilisation coincided with changes in the restriction of movements, which allowed travelling across provinces. During this period, 19.1% of individuals surveyed by Statistics South Africa indicated that they returned to their usual province of residence after the initial lockdown [22]. The change in province of residence is expected as many migrants prefer to retain ties with their households of origin whilst working in other provinces or cities. The impact in mostly rural provinces where poverty is higher is similar to the data from other countries [23]. In Ethiopia and Kenya, while services were interrupted at the start of the pandemic, utilisation of services bounced back in subsequent months, though these analysis did not show the impact by different socioeconomic levels [8,24]. In South Africa, fear of contracting COVID-19 whilst using public transport and in health care facilities was reported which could explain the decline in utilisation [25].

For measles first dose, whilst uptake was interrupted at the onset of the pandemic, the mean change in all facilities increased in the follow-up months except for Eastern Cape and Northern Cape which showed a slight decrease. The most likely reason for this bounce back is the prioritisation of services that require high immunisation coverage to prevent outbreak [26,27]. Early on during the pandemic, field guides were provided by the National Department of Health to outline the process of catch-up of missed vaccinations as well as other child health interventions [28]. Our results were shown to be similar to what was observed in Kenya, Uganda and England where services were interrupted at the start of the pandemic but bounced back in the follow-up months [8,26].

The impact of COVID-19 on severe acute malnutrition was significant at facility level. All quintiles with the exception of quintile 3 showed a significant increase in SAM. The increase in SAM is not surprising as almost 23.6% of South Africans were affected by moderate-to-severe food insecurity in 2020 [29]. For diarrhoea and pneumonia, incidences and deaths in facilities declined when compared to the period before the pandemic. The highest reduction was observed between April and September 2020. The negative impact on diarrhoea was, however, significant only in quintile 1. This observation has been attributed to less time for social interaction in schools and the protective measures against COVID-19 (such as handwashing) which are also effective in reducing some infections. These results are also corroborated by Dorrington et al (2022) in a study that showed a decline in non-COVID-19 mortality in infants and young children [29]. The results are also similar to studies in other countries that have shown a decline in incidences of infectious disease in children [30–33].

Whilst at facility level, antenatal visits, facility delivery in 15- to 19-year-olds, delivery by C-section and maternal mortality increased in absolute and relative numbers, the results were not significant. At the start of the pandemic, national guidelines were provided for care of mothers and new-borns [34,35]. The swift response of government in providing guidance could explain the minimal impact of the pandemic on maternal services.

## Limitations

Our study has strengths and limitations. The main limitation is that we only focused on utilisation in public health facilities. We therefore cannot ascertain whether the change in utilisation for example in wealthier quintiles was due to changes in utilisation in the private sector. The main strength of our study is that we were able to consider equity in utilisation of services through the use of RWI.

## Conclusion

Maternal and child health form part of the quadruple burden of disease in South Africa. Our study shows that COVID-19 disrupted utilisation of child health services. While reduction in child health services at the start of the pandemic was followed by an increase in subsequent months, the recovery was not uniform across different quintiles and geographical areas. This study highlights the disproportionate impact of the pandemic and the need for targeted interventions to improve utilisation of health care services. Early interventions are therefore needed to mitigate disruption of essential services to increase the use of health services such as immunisation during national crises.

## Paper context

Maternal and child health form part of the quadruple burden of disease in South Africa. Currently, the impact of COVID-19 on maternal and child health is not fully documented. This study investigated the indirect effects of COVID-19 on maternal and child health by different geographic areas and relative wealth index. The study highlights the disproportionate impact of the pandemic and the need for targeted interventions to improve utilisation of health care services.
